# Flow cytometry protocol for GLUT4-myc detection on cell surfaces

**DOI:** 10.1042/BSR20231987

**Published:** 2024-04-12

**Authors:** Emilia Zanni-Ruiz, Luis Segundo Mayorga, Martin Alejandro Pavarotti

**Affiliations:** 1 Laboratorio de Transporte Intracelular, Instituto de Histología y Embriología de Mendoza Dr. Mario H Burgos, Mendoza, Argentina; 2 Consejo Nacional de Investigaciones Científicas y Técnicas, Buenos Aires, Argentina; 3 Facultad de Ciencias Exactas y Naturales, Universidad Nacional de Cuyo, Mendoza, Argentina; 4 Universidad Nacional de Cuyo, Mendoza, Argentina

**Keywords:** flow cytometry, GLUT4 translocation, skeletal muscle

## Abstract

Insulin and muscle contraction trigger GLUT4 translocation to the plasma membrane, which increases glucose uptake by muscle cells. Insulin resistance and Type 2 diabetes are the result of impaired GLUT4 translocation. Quantifying GLUT4 translocation is essential for comprehending the intricacies of both physiological and pathophysiological processes involved in glucose metabolism. The most commonly used methods for measuring GLUT4 translocation are the ELISA-type assay and the immunofluorescence assay. While some reports suggest that flow cytometry could be useful in quantifying GLUT4 translocation, this technique is not frequently used. Much of our current understanding of the regulation of GLUT4 has been based on experiments using the rat myoblast cell line (L6 cell) which expresses GLUT4 with a myc epitope on the exofacial loop. In the present study, we use the L6-GLUT4myc cell line to develop a flow cytometry-based approach to detect GLUT4 translocation. Flow cytometry offers the advantages of both immunofluorescence and ELISA-based assays. It allows easy identification of separate cell populations in the sample, similar to immunofluorescence, while providing results based on a population-level analysis of multiple individual cells, like an ELISA-based assay. Our results demonstrate a 0.6-fold increase with insulin stimulation compared with basal conditions. Finally, flow cytometry consistently yielded results across different experiments and exhibited sensitivity under the tested conditions.

## Introduction

The overexpression of fusion proteins with a myc tag is a common technique in cell biology for the identification of intracellular and plasma membrane proteins. This method enables precise and reliable detection of the myc tag, thereby facilitating accurate identification of the target proteins. In this context, L6 cells overexpressing GLUT4-myc have been extensively utilised in studies focusing on GLUT4 translocation and exocytosis [1–4]

The gold-standard techniques for assessing GLUT4 translocation in this normal and similar engineered cell lines have been ELISA-based assays [5–7] and immunofluorescence (IF) [3,8–10], each with its own advantages and limitations.Because of its intrinsic characteristics, the IF assay provides a comprehensive view of cell morphology and intracellular compartments. It additionally facilitates the concurrent localisation of two, three, or more intracellular epitopes [3]. However, observing cellular GLUT4-myc through IF requires epifluorescence microscopy, specific software and an experienced operator to analyse cells individually [3]. Consequently, this last introduces subjectivity due to the operator's prior knowledge. Moreover, detecting and quantifying myc epitope demands a cell-by-cell analysis, requiring the scrutiny of a significant number of cells to acquire statistically reliable outcomes, resulting in a time-consuming process.

In contrast, the ELISA-based assay, commonly used as the main method for identifying GLUT4 translocation, is a cell population technique that enables quick analysis of numerous cells in a short period [4]. The measurements are dependent on the number of cells in each well, which may vary due to cell loss. To address this, replication of each condition is a common practice to obtain more reliable averages. Alternatively, the absorbance results from each well can be normalised by the number of cells per well, and for this purpose, cell counting methods like DAPI nuclear staining (after ELISA assay) combine with an automated cell counting can be employed. However, ELISA-based assays have intrinsic limitations as they do not support additional epitope detection or cell morphology studies.

Flow cytometry (FC), extensively used in immunology for distinguishing and grouping blood cell populations, has numerous applications in cell biology [11]. The FC equipment consists of three main components: the fluidic system, accountable for transferring samples from tubes and creating an exact cell flow; the optical system, which mainly employs lasers, lenses, and filters to gather and transfer light inside the device to the detection system; and the electronic components, which include photodetectors and computers that digitise the photon currents. In essence, FC facilitates a thorough analysis of cells according to their size and intricacy. Additionally, applying protein-specific labelling methods allows for accurate identification and classification of cells based on their protein expression [12].

However, although FC appears promising for measuring GLUT4 translocation, only few studies have used it [13–19] and its effectiveness has not been systematically evaluated. To address this issue, we developed a custom protocol to study the plasma membrane GLUT4myc epitope in L6-GLUT4myc cells in different experimental settings. The results demonstrated the advantage of this assay, including the ability to identify small variations in the kinetics and sensitivity of insulin assays.

## Materials and methods

### Cell culture

Rat L6 myoblasts stably expressing myc-tagged GLUT4 (L6-GLUT4myc) were cultured in α-MEM (GIBCO cat. 11900073) supplemented with 10% FBS and the antibiotics penicillin-streptomycin-amphotericin B (SERENDIPIA Lab, cat. DC2700i and DC2101, respectively) according to the previously established protocol [20]

### Reagents

Human insulin (Humulin R) was obtained from Eli Lilly (Indianapolis, IN, U.S.A.). The analog of adenosine monophosphate 5-Aminoimidazole-4-carboxamide ribonucleotide (AICAR) was obtained from TOCRIS (catalogue #2840) and ionomycin was obtained from Sigma-Aldrich (catalogue #I9657). The primary monoclonal mouse anti-c-Myc antibody (clone 9E10) was purchased from Santa Cruz Biotechnology (Dallas, TX, U.S.A.), and the polyclonal rabbit anti-c-Myc antibody was purchased from Millipore-Sigma (St. Louis, MO, U.S.A.). Secondary antibodies, including goat anti-rabbit IgG H&L (Alexa Fluor^®^ 647) and goat anti-rabbit IgG H&L (Cy3^®^), were purchased from ABCAM, while goat anti-rabbit H&L HRP was from Thermo Scientific (Invitrogen^®^). Sigma FAST OPD (o-phenylenediamine dihydrochloride peroxidase substrate) tablets were purchased from SIGMA-ALDRICH (Catalogue #P9187).

### IF assay

Cell surface GLUT4myc staining was performed according to the method described by Jaldin-Fincatti J.R. in 2017, with some modifications. Briefly, L6-GLUT4myc cells adhered to coverslips were starved of serum for 3 h at room temperature. The cells were then either left untreated (basal condition) or treated with insulin 100 nM for 20 min at 37°C. The cells were then immediately subjected to two cold PBS washes followed by fixation with cold 3% paraformaldehyde (PFA) for 30 min. After fixation, the cells were quenched with two 5-min washes of 0.2 M glycine and blocked for 30 min in PBS containing 2% BSA. For GLUT4myc detection, an anti-c-Myc (9E10) antibody (diluted 1:100) was used for 1 h at room temperature in PBS supplemented with 0.2% BSA. Goat anti-mouse Cy^®^3 conjugate (diluted 1:800) was then applied for 45 min at room temperature in PBS containing 0.2% BSA. After thorough washing, the coverslips were mounted with Mowiol mounting medium. Fluorescence images were captured using an Olympus FV 1000 inverted confocal microscope. Whole cells were scanned along the *z*-axis and a single composite image (collapsed *xy* projection) of the optical sections for each cell was generated using Fiji ImageJ software.

### ELISA-based assay

Surface GLUT4 translocation assays were adapted from previous colorimetric methods with some modifications [21]. These assays were performed using non-permeabilised L6-GLUT4myc myoblasts initially plated in 96-well plates (10,000–15,000 cells per well) with three wells per condition. Briefly, after 3 h of serum starvation, the cells were stimulated with or without 100 nM insulin for 20 min. They were then washed twice with cold PBS containing 1 mM CaCl_2_ and 1 mM MgCl_2_ (pH 7.4). The cells were then fixed with 3% paraformaldehyde for 10 min, quenched with 0.2 M glycine for 5 min and blocked with 5% goat serum for 20 min For GLUT4myc labelling, a solution of rabbit polyclonal anti-c-Myc 1:1000 (0.5 µg/ml) in PBS 5% goat serum was applied and incubated for 50 min at room temperature. After five washes, anti-c-Myc was then detected using an HRP-conjugated goat anti-rabbit antibody (diluted 1:1000) for 40 min at room temperature. Following several washes with PBS, the cells were exposed to OPD solution for 1–4 min and the reaction was stopped by the addition of 3 M of HCl. Absorbance was then measured in a Multiscan Thermo Scientific spectrophotometer at 492 nm, subtracting background absorbance values obtained from cells lacking anti-c-Myc antibody.

### Flow cytometry assay

Cells were plated two days before the assay and allowed to reach 70–85% confluence. On the day of the assay, the cells were washed twice and incubated for 20 min in free calcium-magnesium PBS buffer to facilitate detachment. After rounding, the cells were resuspended at a concentration of 2,000,000 cells/ml in serum-free α-MEM and this cell suspension was divided into 300 µl portions for each condition. After 3 h of serum deprivation, the cell suspension was stimulated by adding 300 µl of α-MEM at twice the concentration of the stimulants, with incubation times as indicated in the corresponding figure legend. For inhibitor conditions, they were added a certain time before the end of serum deprivation. GLUT4 translocation was stopped and the cells were fixed with 2% paraformaldehyde at 4°C for 15 min by adding 600 µl of double concentration (4%) paraformaldehyde. The cell suspension was then centrifuged at 1900 rcf for 4 min and the supernatant was carefully removed, retaining 50 µl of the solution to prevent cell resuspension. The samples were then quenched with 0.1 M glycine for 5 min at room temperature, washed with 1 ml of 5% goat serum in PBS and centrifuged as described above. GLUT4myc was labelled with 150 µl of rabbit polyclonal anti-c-Myc antibody diluted 1:375 in 5% goat serum-PBS (final concentration 1:500) and incubated for 45 min at room temperature with tube inversion every 15 min. Then, 1 ml of 5% goat serum in PBS was added for washing, followed by centrifugation as described above. Rabbit anti-c-Myc antibody was detected using 150 µl of a 1:600 rabbit Alexa 647 secondary solution in 5% goat serum-PBS (final concentration 1:800) for 30 min at room temperature with tube inversion every 10 min. Finally, 1 ml of 5% goat serum in PBS was added for washing, followed by centrifugation as described above, and the remaining volume was adjusted to 200–500 µl. Samples were processed using the BD FACSAria III or ACCURI C6 Plus flow cytometers. A range of between 5,000 and 10,000 events per condition was recorded.

### Data and statistical analysis

**Figure 1:**
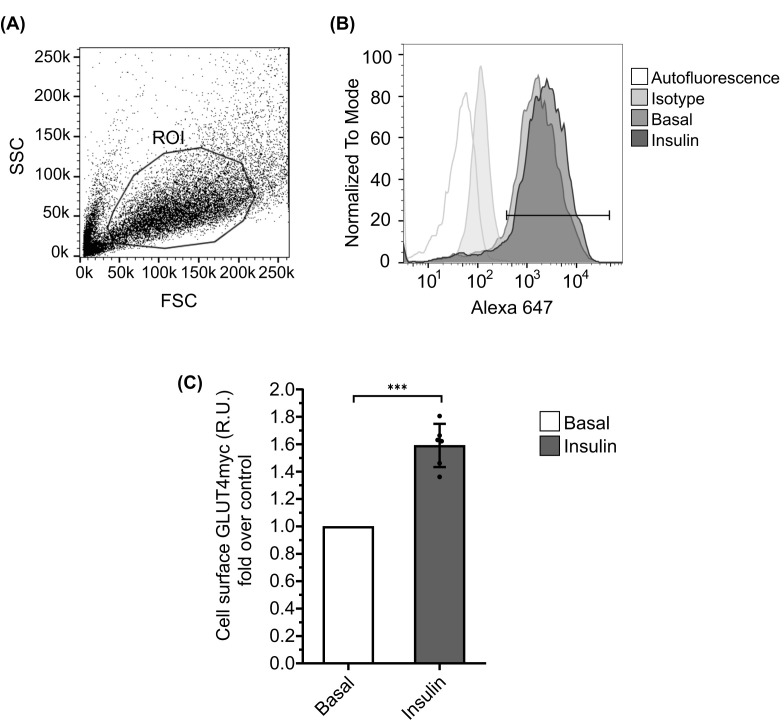
Setup of a flow cytometry-based assay for the quantification of GLUT4 exocytosis (**A**) A total of 20,000 events were recorded for each condition. Based on granularity (SSC-A) and size (FSC-A) parameters, a homogeneous and physiologically relevant population was defined as ‘live cells’ (ROI) containing between 4,000 and 8,000 events. (**B**) Histograms were generated according to Alexa 647 intensity and events within the ROI (white: autofluorescence, light grey: isotype, middle grey: basal and dark grey: insulin). The area under the curve of the specific signal corresponds to the basal treatment (middle grey) and the insulin treatment (dark grey), delimited by a horizontal black line, without including the non-specific signal of the autofluorescence. (**C**) Exocytosis of GLUT4 by flow cytometry. Experiments were normalised to their basal conditions. Bars represent mean ± SEM (*n*=6) ***P<0.0001 calculated by Student’s *t*-test.

**Figure 2:**
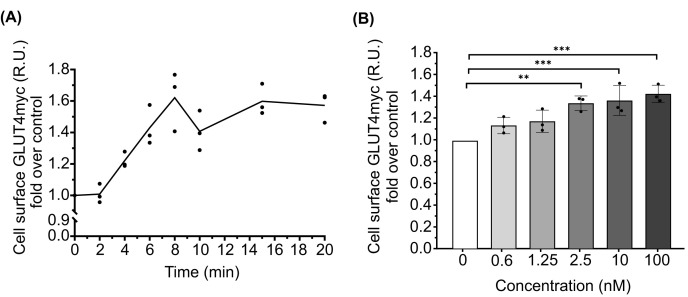
Kinetic and sensitivity assays (**A**) The flow cytometry protocol was subjected to a kinetic assay in L6 GLUT4myc cells. Stimulations were performed at different times (0, 2, 4, 6, 8, 10, 15 and 20 min) with 100 nM insulin (*n*=3). (**B**) On the other hand, the sensitivity protocol was used and stimulation was performed at 10 min with different insulin concentrations (0; 0.6; 1.25; 2.5, 10 and 100 nM). Bars represent mean ± SEM (*n*=3). Each experiment was normalised to its basal condition. **P=0.0017 (2.5 nM); ***P=0.0010 (10 nM); ***P=0.0003 (100 nM) calculated by two-way ANOVA with post-hoc Tukey’s test.

**Figure 3:**
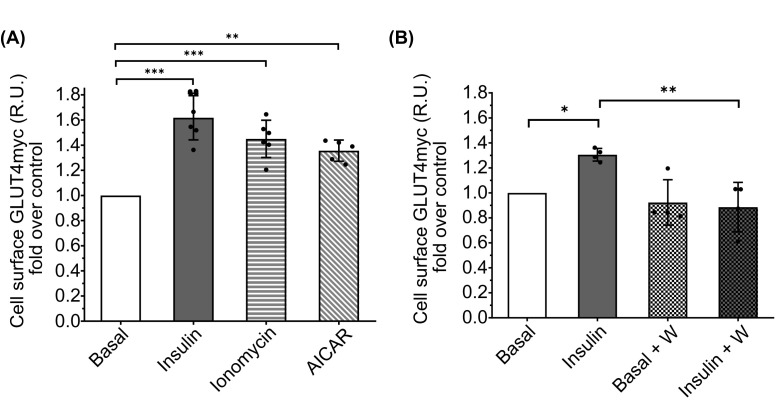
Different stimuli and inhibitions can be measured by flow cytometry (**A**) Stimulation of GLUT4 exocytosis. Different reagents were tested to stimulate exocytosis via different pathways: 100 nM insulin for 20 min, 1 µM ionomycin for 5 min and 2 mM AICAR for 1 hour. Bars represent mean ± SEM (*n*=6). Each experiment was normalised to its basal condition. ***P<0.0001 (insulin), ***P=0.0001 (ionomycin); **P=0.0004 (AICAR). (**B**) Inhibition of GLUT4 exocytosis. Wortmannin (1 µM) was used to inhibit exocytosis 30 min before stimulation with 100 nM insulin for 20 min. Bars represent mean ± SEM (*n*=4). *P=0.0363 (against basal, insulin); **P=0.0046 (against insulin, wortmannin + insulin) calculated by two-way ANOVA with post-hoc Tukey’s test.

**Figure 4:**
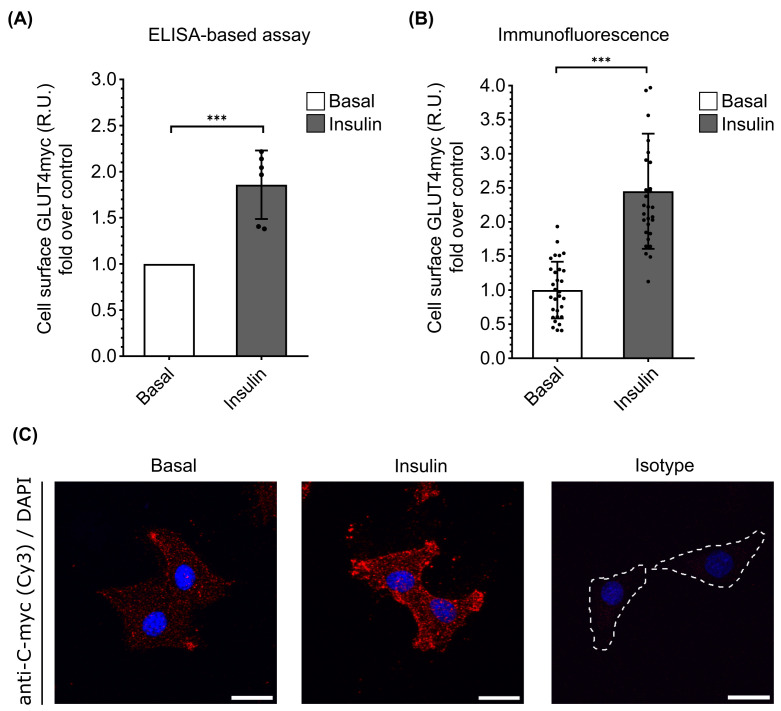
Traditional assays for GLUT4 exocytosis GLUT4 exocytosis was measured by (**A**) ELISA-based assay and (**B**) immunofluorescence. Cells were stimulated with 100 nM insulin for 20 min. Bars represent mean ± SEM, *n*=5 for ELISA-based assay (***P=0.0002) and *n*=30 for immunofluorescence ***P=0.0001 calculated by Student’s *t*-test (15 cells per condition per experiment were evaluated from two independent immunofluorescence). (**C**) Immunofluorescence of surface GLUT4myc in non-permeabilised L6-GLUT4myc cells. GLUT4 (in red) and nuclei (in blue); scale bars: 20 µm.

The data was processed using FlowJo VX. First, a population of interest was defined using the traditional SSC-A (granularity) and FSC-A (size) parameters, corresponding to live cells at the time of the assay. Doublet events were then removed using the FSC-A and FSC-W parameters. Non-specific fluorescence was subtracted from the different conditions assayed. The mean Alexa 647 fluorescence was then determined for each treatment and the values normalised to the basal condition. Raw data for GLUT4myc translocation were converted to fold stimulation over basal. Statistical analyses were performed using paired Student’s *t*-tests on raw data (GraphPad Software, San Diego, CA) for Figures 1 and 4. Two-way ANOVA on raw data with post-hoc Tukey’s tests was used for Figures 2 and 3.

## Results

### Flow cytometry protocol designed and optimised to detect GLUT4-myc on the cell surface

The myoblast L6 cell line, stably overexpressing the GLUT4 protein fused to the first exofacial loop c-myc epitope, has been an essential tool for studying GLUT4 translocation [5,8,22,23]. This pioneering cell line was first created in 1998 by Dr. Klip’s lab [5] and has since become a fundamental component of many groups seeking to unravel the various processes associated with GLUT4 translocation [8,24–28]. As described in the Methods and Materials section, a suspension of L6 GLUT4myc cells is prepared, divided and then exposed to specific treatments. The c-Myc epitope is detected using a primary anti-c-Myc antibody followed by an Alexa-conjugated secondary antibody. The cells are then analyzed by FC and a homogeneous and physiologically relevant population is defined (ROI) (Figure 1A). Figure 1B shows four histograms of autofluorescent, isotype, basal, and insulin-stimulated conditions. The autofluorescence intensity is subtracted for resting and stimulated conditions, and the data are plotted. The increase in cell surface GLUT4myc in response to the stimuli is determined by the increase in fluorescence intensity. Our results show that cell surface GLUT4myc increases by an estimated mean of 0.6-fold compared with basal conditions (Figure 1C)

### Assessing the flow cytometry method with kinetic and sensitivity insulin assays

To assess the reliability of the FC assay, we performed kinetic and sensitivity assays. In the kinetic assay, insulin at 100 nM was applied for different durations (2, 4, 6, 8, 10, 15 and 20 min). The results showed that the increase in GLUT4myc started within 2 min and peaked around 8 min, in agreement with previously published reports [15,29,30]. Similarly, the sensitivity test with increasing insulin concentrations (10 min at 0.6, 1.25, 2.5, 10 and 100 nM) showed increasing levels of GLUT4myc detection starting at 0.6 nM and peaking at 2.5 nM. Both sets of results confirmed that FC is a robust method for detecting subtle differences in cell surface GLUT4myc induced by variations in time and insulin concentration.

### Verification of the flow cytometry method using GLUT4 translocation agonists and inhibitors

To verify the sensitivity of the method, new experiments were performed using the GLUT4 translocation activators ionomycin (Ca^2+^ ionophore) [31,32] and AICAR (AMPK activator) [17,33], and the widely used PI3K inhibitor wortmannin to prevent GLUT4 translocation by insulin [33,34]. Figure 3 shows that FC was able to detect the increase in GLUT4myc at the plasma membrane by ionomycin and AICAR. In comparison, the PI3K inhibitor wortmannin showed a very clear inhibition of GLUT4 translocation by insulin. In addition, the basal conditions preincubated with wortmannin show a tendency towards less GLUT4myc at the plasma membrane, which is consistent with other previous results [35,36], highlighting the sensitivity of flow cytometry in detecting subtle changes in GLUT4 translocation dynamics. Thus, the FC protocol showed reproducibility between assays and comparable results to previously published results using IF and ELISA-based assays.

### Immunofluorescence and ELISA-based assay

To test the main techniques for quantifying GLUT4 translocation, we performed an ELISA-based assay and IF. The experiments were performed under the same conditions as the FC experiment shown in Figure 1C. Figure 4 illustrates the results obtained using the conventional methods of IF and ELISA-based assay. IF was performed in duplicate, with each experiment evaluating 30 cells per condition. In our hands, we obtained an average 1.3-fold increase over basal levels. For the ELISA-based assays, we performed six experiments with three replicates per condition and 12,000 cells per well, showing an average 0.8-fold increase after insulin stimulation.

## Conclusion and discussion

While ELISA and IF assays have traditionally been the most common methods for detecting GLUT4 on the surface, a limited number of studies have explored FC for tracking changes in GLUT4 translocation [16–18]. However, to date, there has been no systematic study using flow cytometry to detect GLUT4 translocation.

In the present study, we developed and optimised an FC protocol to detect GLUT4-myc on the cell surface of the widely used L6-GLUT4myc cell line. The results demonstrate a mean increase of 0.6-fold change in response to insulin compared with basal conditions. While the final result obtained by flow cytometry is lower than that obtained by other methodologies (1.3-fold increase with immunofluorescence and 0.8-fold increase with ELISA-based assays), they consistently exhibit reproducibility across different experiments. This difference could be attributed to geometric changes in cells due to the cell resuspension process, potentially resulting in an increase in cell surface area and a higher basal amount of GLUT4 in suspended cells compared with basal cells adhered to surfaces in IF and ELISA assays.

Our results demonstrate that FC provides a reliable and dynamic approach to assess GLUT4 translocation. We also investigated the sensitivity and versatility of FC by performing kinetic assays, sensitivity tests and experiments with different insulin agonists and inhibitors.

### Advantages of flow cytometry for the assessment of GLUT4 translocation

Among the significant advantages of FC to study GLUT4 translocation, we can highlight a remarkable sensitivity that allows the ability to detect subtle fluctuations in GLUT4 translocation dynamics. The sensitivity assays highlighted the ability of FC to detect changes in GLUT4myc levels at low concentrations of insulin, 1.25 nM, with a peak response at 10 nM. In addition, this high sensitivity was clearly demonstrated in our kinetic assays, where we observed a rapid increase in GLUT4myc on the cell surface as early as 2 min after insulin stimulation.

### Versatility of flow cytometry in GLUT4 research

Our study also extended the utility of FC in GLUT4 research by evaluating its performance with different insulin agonists and inhibitors. Here, we have shown that FC is a versatile tool to detect changes in GLUT4myc translocation induced either by the GLUT4 translocation triggers, the calcium ionophore ionomycin or the AMPK agonist AICAR, and the down-regulation achieved by inhibitors like the PI3kinese inhibitor. These results highlight the adaptability of FC to study GLUT4 translocation under different experimental conditions.

### Comparison with other methods

As mentioned above, flow cytometry (FC) offers several advantages over other conventional techniques for studying GLUT4 translocation. While immunofluorescence (IF) provides insight into cell morphology and intracellular compartments, it requires epifluorescence microscopy, specialised software and subjective operator expertise. On the other hand, ELISA-based assays offer high throughput but can be prone to errors associated with cell seeding and population-based measurements. Thus, in comparison to IF, cytometry results provide the advantage of analyzing cell populations and, unlike ELISA assays, allow for the selection of phenotypically normal cells and the incorporation of additional labeling. Hence, FC bridges these gaps by providing a robust, sensitive, high-throughput method capable of providing both population-based data and single-cell analysis.

Regarding previous FC methods used to observe GLUT4 translocation, this cytometry method has been specifically developed for the L6 GLUT4myc cell line, which is recognised for its stable expression of the glucose transporter. This cell line has undergone thorough validation and has been utilised in conjunction with various methodologies to investigate events and processes associated with GLUT4 exocytosis. Unlike previous studies using FC, none carried out the study with L6 GLUT4myc cells. Instead, they utilised 3T3 fibroblasts, adipocytes derived from this cell line, and primary cardiomyocytes. Moreover, they employed different techniques to express and detect GLUT4, as well as different primary antibodies. These dissimilarities make it challenging to compare their findings to ours.

Our study confirms that FC is not only a reliable alternative to traditional methods but also a valuable tool for expanding the scope of GLUT4 translocation studies in L6 GLUT4myc cells.

In summary, the FC method presented here provides an effective and sensitive approach to studying GLUT4 translocation at the cell surface. Using FC, we can detect subtle changes in GLUT4 dynamics in response to different stimuli, providing a valuable tool to better understand the underlying mechanisms of glucose uptake regulation. Its ability to rapidly and accurately analyze large numbers of cells makes it a promising choice for studies of GLUT4 translocation compared with traditional methods such as ELISA and immunofluorescence.

## Data Availability

Any materials, data, code and protocols used in this work are specifically detailed in the manuscript.

## Abbreviations

FC: flow cytometry
IF: immunofluorescence
PFA: paraformaldehyde
AICAR: 5-Aminoimidazole-4-carboxamide ribonucleotide
